# The 50 Most-cited Articles in Gastroenterology and Hepatology from Mainland China

**DOI:** 10.12669/pjms.331.12286

**Published:** 2017

**Authors:** Sun-kuan Hu, Jie Huang, Wan-dong Hong, Xiao-jing Du, Rong Jin, Tie-su Lin

**Affiliations:** 1 Sun-kuan Hu, Dept. of Gastroenterology and Hepatology, Dept. of Epidemiology, The First Affiliated Hospital of Wenzhou Medical University, Wenzhou 325000, Zhejiang Province, P.R. China; 2 Jie Huang, Academic Affair Office, The First Affiliated Hospital of Wenzhou Medical University, Wenzhou 325000, Zhejiang Province, P.R. China; 3 Wan-dong Hong, Dept. of Gastroenterology and Hepatology, The First Affiliated Hospital of Wenzhou Medical University, Wenzhou 325000, Zhejiang Province, P.R. China; 4 Xiao-jing Du, Dept. of Gastroenterology and Hepatology, Dept. of Epidemiology, The First Affiliated Hospital of Wenzhou Medical University, Wenzhou 325000, Zhejiang Province, P.R. China; 5 Rong Jin, Dept. of Epidemiology, The First Affiliated Hospital of Wenzhou Medical University, Wenzhou 325000, Zhejiang Province, P.R. China; 6 Tie-su Lin, Dept. of Gastroenterology and Hepatology, Dept. of Epidemiology, The First Affiliated Hospital of Wenzhou Medical University, Wenzhou 325000, Zhejiang Province, P.R. China

**Keywords:** Science Citation Index, Gastroenterology and Hepatology, Mainland China, Bibliometrics, Web of Science

## Abstract

**Objective:**

To identify and analyze the 50 most-cited gastroenterology and hepatology articles originating from mainland China.

**Methods:**

We utilized the 2015 edition of Journal Citation Reports and PubMed to determine the 50 most-cited gastroenterology and hepatology articles from 75 professional journals and four leading journals in clinical medicine, which are The New England Journal of Medicine, The Lancet, The Journal of the American Medical Association, and The British Medical Journal. Then we excluded the articles written outside mainland China and collected the basic information, including the title, authors, year of publication, source journal, city, institution, number of citations, and topic of the research.

**Results:**

The number of citations for the top 50 papers ranged from 279 to 89 (mean, 129). These articles were published between 2005 and 2012, in which 2009 was the year with the largest number of highly cited papers(13). All articles were published in 15 journals. The journal Hepatology published the largest number of articles(21), followed by Journal of Gastroenterology and Hepatology(4), Journal of Hepatology(4) and World Journal of Gastroenterology(4). The top 50 articles originated mainly from Shanghai(20), Guangzhou(13) and Beijing(6). Sun Yat-sen University produced most highly cited papers(10). The number of basic research was far more than clinical research, of which the ratio was about 1.78(32:18). In all these articles, hepatocellular carcinoma was the most-discussed topic(19), followed by hepatitis B virus(8) and endoscopic(5).

**Conclusions:**

Although a large gap remains between mainland China and the global community, the gastroenterology and hepatology research from China is gradually recognized by the world.

## INTRODUCTION

With a population of over 1.35 billion, China is the world’s most populous country and the second-largest country by land area, covering approximately 9.6 million square kilometers. Since the introduction of economic reforms in 1978, China has become one of the world’s fastest-growing major economies and has been the second-largest economy.

At the same time, China’s government funding is increasing by 20% a year, which stimulates research and development.^1^ Together with other great motive power, China is now ranked as the highest producing country for scientific research publications since 2012.^2^ In addition, there has been rapid development in the biomedical fields.^3^-^5^ Such development has also appeared in the field of gastroenterology and hepatology, so the 2013 World Congress of Gastroenterology was held in China and more and more researchers from mainland China show their academic achievements on the international stage. Therefore, China is becoming a leading force in medical research, including gastroenterology and hepatology.

However, the impact of gastroenterology and hepatology studies from mainland China has not been investigated so far to the best of our knowledge. Since the advent of bibliometric science, citation analysis has been widely used to evaluate the influence of a scientific article, which focuses on the methodological, quality and ranking issues of authors, journals, institutions and nations.^6^ Citation analysis has been performed in otolaryngology–head and neck surgery,^7^ critical care medicine,^8^ obstetrics and gynecology.^9^ cardiac surgery,^10^ orthopaedic surgery,^5^,^11^,^12^ radiology^13^ and acute pancreatitis.^14^ Because acute pancreatitis is only one of the most common diseases in gastroenterology, so there were no gastroenterology and hepatology articles from China included in previous “most-cited” studies, and we believe perhaps no study has been performed specifically to analyze the most-cited papers in gastroenterology and hepatology from mainland China.

We therefore sought to (1) identify the 50 most-cited gastroenterology and hepatology articles originating in mainland China and (2) analyze these articles in terms of source journals, institutions and topics.

## METHODS

A search was performed on March 30, 2015, using the bibliometric database Web of Science (Thomson Reuters, Philadelphia, PA, USA), a method that has been used in similar studies.^5^,^14^-^17^ There were 75 journals under the subject category of “gastroenterology & hepatology” in *Journal Citation Report* for 2015. The four leading journals in clinical medicine were also searched, *The New England Journal of Medicine, The Lancet, The Journal of the American Medical Association*, and *The British Medical Journal*. Then articles (1964-2014) from the 79 journals were ranked based on the number of citations. Using a previous protocol from similar studies,^5^,^14^ to exclude articles written outside mainland China, a filter of “Countries/territories” was applied first by choosing “Peoples R China” in searching. To exclude other document types, “Article” was chosen. Using the platform of Pubmed, each article was evaluated and those without a primary address or a reprint address from mainland China were excluded.

Following the methods of previous studies,^5^,^14^ basic information was collected, including the title, authors, year of publication, source journal, city, institution, number of citations, and topic of the research.

## RESULTS

The 50 articles are listed in Table-I in descending order, based on the number of citations they received. The most cited article received 279 citations, and the least-cited article received 89 citations. The mean number of citations per article was 129.

**Table-I:** The top 50 articles in Gastroenterology and Hepatology from mainland China.

Rank	Article	Number of Citations
1	Increased regulatory T cells correlate with CD8 T-cell impairment and poor survival in hepatocellular carcinoma patients	279
2	Directed differentiation of human embryonic stem cells into functional hepatic cells	272
3	Effect of microRNA-29 on apoptosis, tumorigenicity, and prognosis of hepatocellular carcinoma	237
4	Differential expression of microRNA species in human gastric cancer versus non-tumorous tissues	235
5	Increased intratumoral IL-17-producing cells correlate with poor survival in hepatocellular carcinoma patients	201
6	Interleukin-17-producing CD4(+) T cells increase with severity of liver damage in patients with chronic hepatitis B	193
7	MicroRNA-195 suppresses tumorigenicity and regulates G1/S transition of human hepatocellular carcinoma cells.	190
8	Prevalence of and risk factors for fatty liver in a general population of Shanghai, China	188
9	Identification of functional genetic variants in cyclooxygenase-2 and their association with risk of esophageal cancer	170
10	Up-Regulated MicroRNA-143 Transcribed by Nuclear Factor kappa B Enhances Hepatocarcinoma Metastasis by Repressing Fibronectin Expression	162
11	Bonemarrow-derived mesenchymal stem cells protect against experimental liver fibrosis in rats	160
12	Lamivudine in late pregnancy to prevent perinatal transmission of hepatitis B virus infection: a multicentre, randomized, double-blind, placebo-controlled study	143
13	Indications and detection, completion, and retention rates of small-bowel capsule endoscopy: a systematic review	137
14	Geographic distribution, virologic and clinical characteristics of hepatitis B virus genotypes in China	132
15	Risk factors of pancreatic leakage after pancreaticoduodenectomy	132
16	Survival prediction of gastric cancer by a seven-microRNA signature	131
17	The emergence of inflammatory bowel disease in the Asian Pacific region	128
18	Down-Regulated MicroRNA-152 Induces Aberrant DNA Methylation in Hepatitis B Virus-Related Hepatocellular Carcinoma by Targeting DNA Methyltransferase 1	126
19	Telbivudine versus lamivudine in Chinese patients with chronic hepatitis B:Results at 1year of a randomized, double-blind trial	120
20	Prevention of hepatitis B recurrence after liver transplantation using lamivudine or lamivudine combined with hepatitis B Immunoglobulin prophylaxis	119
21	miR-15b and miR-16 are implicated in activation of the rat hepatic stellate cell: An essential role for apoptosis	116
22	The putative tumour suppressor microRNA-124 modulates hepatocellular carcinoma cell aggressiveness by repressing ROCK2 and EZH2	114
23	IFN-gamma-induced TNFR2 expression is required for TNF-dependent intestinal epithelial barrier dysfunction	111
24	Long Noncoding RNA High Expression in Hepatocellular Carcinoma Facilitates Tumor Growth Through Enhancer of Zeste Homolog 2 in Humans	110
25	Prediction of significant fibrosis in HBeAg-positive patients with chronic hepatitis B by a noninvasive model	109
26	Risk Factors for ERCP-Related Complications: A Prospective Multicenter Study	108
27	MicroRNA-125b Suppressesed Human Liver Cancer Cell Proliferation and Metastasis by Directly Targeting Oncogene LIN28B	106
28	Involvement of PI3K/PTEN/AKT/mTOR pathway in invasion and metastasis in hepatocellular carcinoma: Association with MMP-9	106
29	Hepatology - Microarray analysis of microRNA expression in hepatocellular carcinoma and non-tumorous tissues without viral hepatitis	103
30	Expression and Functional Significance of Twist1 in Hepatocellular Carcinoma: Its Role in Vasculogenic Mimicry	103
31	Initial study of microRNA expression profiles of colonic cancer without lymph node metastasis	102
32	Circulating miR-221 directly amplified from plasma is a potential diagnostic and prognostic marker of colorectal cancer and is correlated with p53 expression	101
33	Meta-analysis of endoscopic submucosal dissection versus endoscopic mucosal resection for tumors of the gastrointestinal tract.	101
34	Low central venous pressure reduces blood loss in hepatectomy	101
35	Fatty live rand the metabolic syndrome among Shanghai adults	99
36	Effects of nonalcoholic fatty liver disease on the development of metabolic disorders	98
37	MicroRNA-30d Promotes Tumor Invasion and Metastasis by Targeting Galphai2 in Hepatocellular Carcinoma	98
38	A retrospective study of the application on double-balloon enteroscopy in 378 patients with suspected small-bowel diseases	97
39	Expression of hypoxia-inducible factor 1 alpha and vascular endothelial growth factor in hepatocellular carcinoma: Impact on neovascularization and survival	96
40	MicroRNA-101 Regulates Expression of the v-fos FBJ Murine Osteosarcoma Viral Oncogene Homolog (FOS) Oncogene in Human Hepatocellular Carcinoma	96
41	Activated Monocytes in Peritumoral Stroma of Hepatocellular Carcinoma Promote Expansion of Memory T Helper 17 Cells	95
42	Role of Overexpression of CD151 and/or c-Met in Predicting Prognosis of Hepatocellular Carcinoma	95
43	A prospective and open-label study for the efficacy and safety of telbivudine in pregnancy for the prevention of perinatal transmission of hepatitis B virus infection	94
44	Performance of the Aspartate Aminotransferase-to-Platelet Ratio Index for the Staging of Hepatitis C-Related Fibrosis: An Updated Meta-Analysis	93
45	Liver-Enriched Transcription Factors Regulate MicroRNA-122 That Targets CUTL1 During Liver Development	92
46	Diagnostic yield and therapeutic impact of double-balloon enteroscopy in a large cohort of patients with obscure gastrointestinal bleeding	91
47	Hepatitis B Virus X Protein Sensitizes Cells to Starvation-Induced Autophagy via Up-regulation of Beclin 1 Expression	91
48	Lentiviral-Mediated miRNA Against Osteopontin Suppresses Tumor Growth and Metastasis of Human Hepatocellular Carcinoma	90
49	MicroRNA-29b Suppresses Tumor Angiogenesis, Invasion, and Metastasis by Regulating Matrix Metalloproteinase 2 Expression	89
50	Functional Linkage of Cirrhosis-Predictive Single Nucleotide Polymorphisms of Toll-like Receptor 4 to Hepatic Stellate Cell Responses	89

**Fig. 1:**
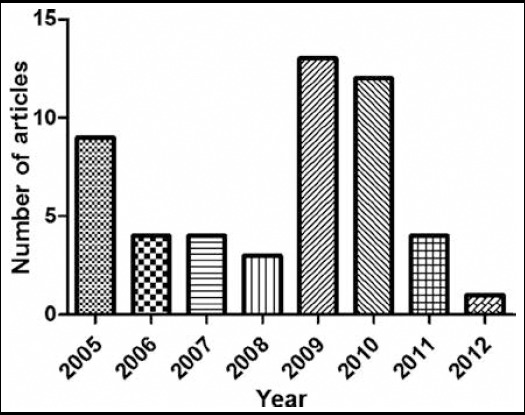
The number of 50 top-cited articles in gastroenterology and hepatology produced from 2005 to 2012.

These articles were published from 2005 to 2012. There were 9 oldest cited articles published in 2005. The newest article was published in 2012 and was written by Zheng Fang. 2009 was the year with the largest number of highly cited papers were published(n=13, 26%), followed by 2010(n=12, 24%). Although there were only 4 articles published in 2007, the most cited two articles were produced.(Fig.1)

The 50 top-cited articles were published in 15 journals, led by Hepatology(21 articles), followed by Journal of Gastroenterology and Hepatology(4 articles), Journal of Hepatology(4 articles) and World Journal of Gastroenterology(4 articles).(Table-II)The top 50 articles originated from 13 Chinese cities, with Shanghai producing 20 articles, followed by Guangzhou with 13 and Beijing with 6, which were far more than other cities.

**Table-II:** Journals publishing gastroenterology and hepatology articles by authors from mainland China.

Journal	Number of articles	5-YearImpact factor
Hepatology	21	11.19
Journal of Gastroenterology and Hepatology	5	3.627
Journal of Hepatology	4	10.401
World Journal of Gastroenterology	4	2.433
Gastroenterology	3	13.926
Gut	2	13.319
American Journal of Gastroenterology	2	9.213
Endoscopy	2	5.196
Journal of Viral Hepatitis	2	3.307
Gastrointestinal Endoscopy	1	4.9
Liver Transplantation	1	3.793
Current Opinion in Gastroenterology	1	3.664
Hepatology Research	1	2.218
Journal of Digestive Diseases	1	1.924

Altogether, 27 institutions produced these 50 top-cited articles. Institutions associated with more than one paper were Sun Yat-sen University(n=10; 20%), Shanghai Jiao tong University(n=6; 12%), Second Military Medical University(n=5; 10%), Fudan University(n=4; 8%), Peking University(n=2, 4%), and Shanghai Second Medical University(n=2; 4%).(Table-III)

**Table-III:** Institutions associated with more than one article.

Institution(city)	Number of articles
Sun Yat-sen University(Guangzhou)	10
Jiao Tong University(Shanghai)	6
Second Military Medical University(Shanghai)	5
Fudan University(Shanghai)	4
Peking University(Beijing)	2
Shanghai Second Medical University(Shanghai)	2

The top first author was Fan Jian-Gao with three publications on the list, meanwhile the other authors were all with one article. Of the top 50 articles, 32 reported basic research and 18 were clinical studies. Hepatocellular carcinoma was the most-discussed topic(n=19, 38%), followed by hepatitis B virus(n=8, 16%), endoscopic(n=5, 10%).(Table-IV)

**Table-IV:** Classification of research by topic.

Topic	Number of articles
Hepatocellular Carcinoma	19
Hepatitis B Virus	8
Endoscopic	5
Fatty liver	3
Hepatic Fibrosis	3
Colorectal Cancer	2
Gastric Cancer	2
Esophageal Cancer	1
Hepatectomy	1
Hepatitis C Virus	1
Human Embryonic Stem Cells	1
Inflammatory Bowel Disease	1
Intestinal Epithelial Barrier Dysfunction	1
Liver Development	1
Pancreaticoduodenectomy	1

## DISCUSSION

There is no doubt that citation analysis can supply quantitative information about journals, institutions, authors which is helpful to identify classic works and high-impact journals. It can also help us recognize important advances in research and add useful perspective on historical developments in our field.^14^ Although citation analysis of the top cited articles has been performed in multiple medical fields,^5^,^7^-^10^,^13^,^14^ to the best of our knowledge, this is the first bibliometric analysis to reveal the top citations from mainland China in the field of research about gastroenterology and hepatology.

**Fig. 2:**
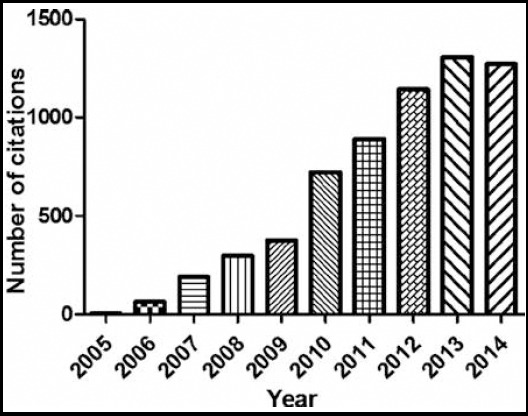
The total number of citations of 50 top-cited articles in gastroenterology and hepatology in every year.

There were limitations to our study. One was that according to the well-defined method, this study did not include gastroenterology subject articles in non-gastroenterology journals, a potential source of numerous highly cited sources. Textbooks and monographs were also not included, and although often not a source of original material, they are frequently cited.^7^ Moreover, although the well-defined method was used to identify influential articles, important ones from mainland China that have been cited less often were not included. And the number of citations that an article receives is not necessarily a reflection of the quality of research.^5^ Finally, using total number of citations as a standard of impact would be expected to favor older articles that accumulate a larger number of citations with time; however, we found it interesting and somewhat counterintuitive that the most recent decade in our search garnered the largest number of top-cited articles.^5^ In general, as shown in Fig.2, the total number of citations of 50 top-cited articles in gastroenterology and hepatology from mainland China in every year is gradually rising. This reflects that research coming from mainland China is gaining visibility and impact in more recent years.

The top 50 articles were cited between 89 and 279 times, which are obviously lower than those of global gastroenterology and hepatology research field, where the citation numbers were between 1383 and 4827. Based on this data, it is not surprising that there was no Chinese article appearing in the 50 top-cited articles list. Although these Chinese articles were not as approbatory as the ones from other countries by experts in gastroenterology and hepatology, they were recognized by the authoritative journals. It is no doubt that Gastroenterology, Gut and Hepatology are journals with most high impact factor, which published 52% (n=26) of the 50 top-cited articles from mainland China. (Table-II) However, there were 86% (n=43) of the top-cited articles from global community published in these three journals, which shows that there is still a need to improve the quality of Chinese research.

Thirteen cities contributed to the top 50 list of highest cited articles, led by Shanghai, Guangzhou and Beijing, and of which the number was far more than other cities. This finding confirms the cities’ overwhelming impact on medical science research because of its large population and the abundant financial resources available to the scientific community.^14^

Our top-50 list included more basic research articles than clinical studies, of which the ratio was approximately 1.78(32:18). This ratio was much more higher than the average ratio (1.31:1, range 0.7:1-1.5:1) of Chinese clinical research,^3^ caused by several reasons. Firstly, it is influenced by the characteristic of clinical studies, which needs long time treatment and complex follow up. Secondly, compared to western-style health-care^18^ system, it is lack of sound information and not convenience in mainland China, which has impact on the quality of clinical subjects. Finally, insufficient funding, lack of available time, an unsupportive research environment, and a deficient clinical research teaching program are also important and complex factors leading to fewer clinical studies.19

Of the 50 top-cited articles, Hepatocellular carcinoma and hepatitis B virus related articles accounted for more than 50%(27/50). This may be connected with the high incidence rate of these diseases. In China, there were approximately 93 million people infected with HBV, which was indicated by an epidemiologic serosurvey of hepatitis B that the weighted prevalence of HBsAg in the Chinese population aged 1-59 years was 7.2%.^20^,^21^ The spectrum of disease and natural history of chronic HBV infection are diverse and variable, ranging from an inactive carrier state to progressive chronic hepatitis B (CHB), which may evolve to cirrhosis and hepatocellular carcinoma (HCC).^22^ All over the world, approximately 75% of liver cancer occurs in Asia, with China accounting for more than 50% of the world’s burden.^23^,^24^ Fortunately, the incidence of hepatitis B virus is decreasing gradually in China, which is because of programs to reduce aflatoxin B1 (AFB1) exposure and hepatitis B virus(HBV) transmission and other public health efforts.^25^ These are strong indications that Chinese medical workers in gastroenterology and hepatology not only give their contributions to preventing and curing hepatocellular carcinoma and hepatitis B virus, but also their related studies are recognized by the world.

In addition, there were five articles about endoscopic and published in Endoscopy, American Journal of Gastroenterology, and Gastrointestinal Endoscopy, which improved that much progress in GI endoscopy has been made by Chinese endoscopists, the efforts and achievements have gradually gained international recognition.

Citation frequency is by no means a perfect measure of scientific impact, but considered as one of several valid and legitimate indicators in identifying classic work. Compared to the global community, the gastroenterology and hepatology research in mainland China remains having lower impact. But the authors of these top-cited 50 articles should be proud of their achievements, they were the representative of efforts made in gastroenterology and hepatology of mainland China in recent 60 years, which was gradually being recognized by the world.
